# RUNX1, a new regulator of EMT in breast cancer

**DOI:** 10.18632/oncotarget.15623

**Published:** 2017-02-20

**Authors:** Saleh Khawaled, Rami I. Aqeilan

**Affiliations:** Lautenberg Center for Immunology and Cancer Research, IMRIC, Hebrew University-Hadassah Medical School, Jerusalem

**Keywords:** RUNX, metastasis, EMT, TGF-β, breast cancer

Breast cancer is the most common cancer among women, and the second leading cause of deaths due to cancer in the US [1]. About 90% of deaths from cancer are due to metastasis, a complex process in which tumor cells escape the primary local tumor, invade through surrounding tissues to spread and colonize distant organs. Tumor cells frequently undergo epithelial-to-mesenchymal transition (EMT), a process whereby cells downregulate the epithelial specific adherens junction proteins including E-cadherin and re-express mesenchymal markers such as Vimentin and N-cadherin. As a result of this transition, the mesenchymal cells migrate from the epithelial layer and invade distant areas of the body [2]. Therefore, molecular characterization of EMT modulators could be a significant step toward better understanding and restraining of metastasis. In this issue, Hong and colleagues demonstrate a novel function of RUNX1 in sustaining mammary normal epithelial morphology and preventing EMT, and suggest that RUNX1 levels could be a prognostic indicator of breast cancer progression [3].

RUNX transcription factors are essential regulators of diverse developmental processes, with roles in proliferation, differentiation, apoptosis and cell lineage specification. In mammals, there are three *RUNX* genes:* RUNX1, RUNX2* and *RUNX3*, each with distinct tissue specific expression patterns. RUNX1 is an extensively studied transcription factor which is well known in regulating development and maintenance of mammalian hematopoiesis and in translocation mediated leukemias’ development (reviewed in [4]). Nevertheless, emerging functions of Runx1 in solid tumors have been recently shown. Of particular interest, Runx1 is the predominant RUNX family member expressed in human breast epithelial cells [5]. The most compelling evidence for its role in breast cancer is the occurrence of *RUNX1* somatic mutations in breast tumors [6].Furthermore, RUNX1 is one of the 17 genes whose expression pattern predicts breast cancer metastasis; its expression is less abundant in breast cancer as compared to normal breast epithelial cells, and it progressively decreases with increasing breast cancer aggression [7, 8].

In their latest study, Hong and coworkers identified a novel role of Runx1 in regulating breast cancer progression [3]. Initially, authors demonstrated that Runx1 expression is reduced in a number of breast cancer cell lines. In particular, Runx1 levels were low in tumorigenic MCF10A derivative cells relative to its high levels in the parent MCF10A, a normal-like mammary epithelial cell. Furthermore, decreased Runx1 expression was associated with EMT markers. This led the authors to hypothesize that Runx1 might act as a tumor suppressor and that its loss is coupled with EMT and perhaps tumor progression. Careful analysis of this assumption demonstrated that induction of EMT through activation of the TGF-β-Smad pathway or by serum deprivation is associated with reduced Runx1 levels. The detailed mechanism by which EMT mediates downregulation of RUNX1 is still unknown. Furthermore, manipulation of RUNX1 expression in breast cancer cells was enough to determine their epithelial/mesenchymal phenotype. In particular, depletion of *RUNX1*, mimicking the situation in breast cancer, led to initiation of EMT: change of morphology toward spindle shape, loss of epithelial marker E-cadherin along with up-regulation of mesenchymal markers including Vimentin and N-cadherin. At the molecular level, authors performed transcriptomic analysis and ChIP-qPCR assays to reveal that Runx1 serves as an EMT downstream transcription factor mediating TGFβ signaling. Finally, authors evaluated RUNX1 expression in breast cancer samples and found that its level is significantly reduced in breast cancer cells that metastasize to lymph nodes relative to the primary tumor site highlighting its prognostic value.

**Figure 1:**
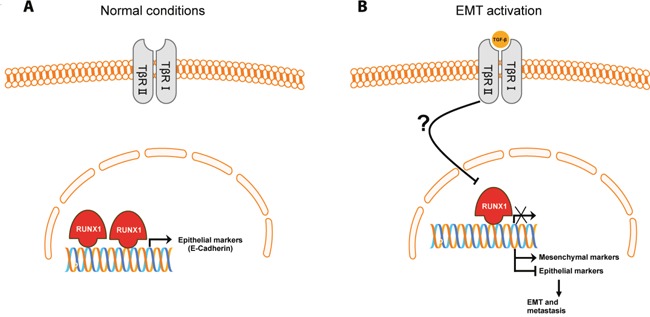
EMT activation inhibits Runx1-mediated mammary epithelial phenotype **A.** In normal mammary conditions RUNX1 promotes transcription of epithelial markers, such as E-cadherin, and thus preserve a mammary epithelial cell phenotype. **B.** Activation of the EMT process either by TGF-β ligands or serum deprivation, leads to reduced Runx1 levels, by an unknown mechanism, and transcription of mesenchymal markers and hence inhibition of the epithelial markers, leading to epithelial-to-mesenchymal transition (EMT) and breast cancer progression.

Altogether, the study by Hong et al underscores the critical function of Runx1 as a key transcription factor in regulating EMT and cell morphology in breast cancer (Figure 1). The study pave the way toward future analysis of the effect of specific targeted deletion of Runx1 in mammary cancer mouse models and assessment of its therapeutic potential for breast cancer intervention.
